# Microarray Analysis of Tomato’s Early and Late Wound Response Reveals New Regulatory Targets for Leucine Aminopeptidase A 

**DOI:** 10.1371/journal.pone.0077889

**Published:** 2013-10-24

**Authors:** Melissa A. Scranton, Jonathan H. Fowler, Thomas Girke, Linda L. Walling

**Affiliations:** Department of Botany and Plant Sciences, Center for Plant Cell Biology, University of California Riverside, Riverside, California, United States of America; University of California, Irvine, United States of America

## Abstract

Wounding due to mechanical injury or insect feeding causes a wide array of damage to plant cells including cell disruption, desiccation, metabolite oxidation, and disruption of primary metabolism. In response, plants regulate a variety of genes and metabolic pathways to cope with injury. Tomato (*Solanum lycopersicum*) is a model for wound signaling but few studies have examined the comprehensive gene expression profiles in response to injury. A cross-species microarray approach using the TIGR potato 10-K cDNA array was analyzed for large-scale temporal (early and late) and spatial (locally and systemically) responses to mechanical wounding in tomato leaves. These analyses demonstrated that tomato regulates many primary and secondary metabolic pathways and this regulation is dependent on both timing and location. To determine if LAP-A, a known modulator of wound signaling, influences gene expression beyond the core of late wound-response genes, changes in RNAs from healthy and wounded *Leucine aminopeptidase A*-silenced (*LapA-SI*) and wild-type (WT) leaves were examined. While most of the changes in gene expression after wounding in *LapA-SI* leaves were similar to WT, overall responses were delayed in the *LapA-SI* leaves. Moreover, two *pathogenesis-related 1* (*PR-1c* and *PR-1a2*) and two *dehydrin* (*TAS14* and *Dhn3*) genes were negatively regulated by LAP-A. Collectively, this study has shown that tomato wound responses are complex and that LAP-A’s role in modulation of wound responses extends beyond the well described late-wound gene core.

## Introduction

 In nature, plants must cope with a multitude of stresses individually and simultaneously. Many of the abiotic (rain, hail, wind) and biotic stresses (herbivory) breach cellular integrity causing membrane disruption, desiccation, lipid and protein oxidation, and protein aggregation [1]. This damage can range from mild (responses to phloem-feeding whiteflies, psyllids and aphids) to extreme (responses to pruning, hail or herbivores that chew and tear plant tissues) [2]. A plant’s ability to rapidly respond to its injured status is integral to activating and modulating the pathways to promote cellular healing, limit pathogen ingression into wound sites and interfere with herbivore success [3-5]. 

 At the core of the wound response are the defenses activated by oxylipins, including jasmonic acid (JA) and its bioactive isoleucine conjugate JA-Ile [6]. Many of the JA-regulated genes encode proteins that directly interfere with insect performance by increasing anti-nutritive proteins and chemicals or are involved in the emission of volatile organic compounds to attract natural enemies to herbivore-infested plants [7,8]. While oxylipin-regulated insect defenses are often the primary response, many other defenses are required to protect against the wide array of abiotic and biotic stresses associated with wounding [9-11]. Therefore, the wound-response pathway involves the integration of a complex and dynamic defense-signaling network that involves JA, salicylic acid (SA), abscisic acid (ABA), ethylene (ET), gibberellic acid (GA), brassinosteroids, cytokinins, as well as reactive oxygen species (ROS) and redox changes [12]. 

 Insights into the dynamics and specificity of damage-induced responses have been gleaned from small- and large-scale microarray studies of wounding and herbivory in *Arabidopsis* and poplar [9-11,13,14]. While *Solanum lycopersicum* (tomato) and *Nicotiana attenuata* have served as model organisms to understand wound responses in the Solanaceae [15,16], large-scale microarray studies focused on understanding recognition of self-damage are few. Microarray studies of the Solanaceae have primarily used small-scale oligonucleotide arrays focused on a narrow set of core defense genes to study herbivory, wounding, or methyl jasmonate (MeJA) treatments [17-19]. Two studies have used large-scale arrays to study responses to the oral secretions of the Colorado potato beetle [20] and treatments with MeJA, JA’s biosynthetic precursors or coronatine [21]. 

 While there is substantive overlap between JA-induced defenses and injury, there may be critical distinctions in plant responses to mechanical damage including JA-independent responses [9,19,22,23]. Furthermore, given tomato’s seminal role in understanding JA-signaling and identifying a core of defenses associated with herbivory, it is timely to examine changes RNA levels that occur when plants perceive injury. Based on studies with well characterized wound-response genes, mechanically damaging tomato leaves results in temporal (early and late) and spatial (local and systemic) changes in gene expression [15]. The early wound-response gene RNA levels are up-regulated 0.5 to 2 hr after injury and are often involved in amplification of the octadecanoid pathway. The late wound-response gene RNA levels increase from 4 to 24 hr and many encode proteins with anti-nutritive roles including: polyphenol oxidase (PPO), the serine proteinase inhibitors (PinI and PinII), arginase, and threonine deaminase [7]. 

 The acidic leucine aminopeptidase (LAP-A) is a late wound-response protein present in a subset of the Solanaceae [24] and has an important role in insect defense. Silencing of *Lap* genes in tomato and *Solanum nigrum* (nightshade) leads to increases in plant susceptibility to caterpillars and larger insect masses [25,26]. Reciprocally, transgenic tomatoes that ectopically express the tomato *LapA1* gene (*LapA-OX*) are more resistant to insect feeding and delays in insect growth and development are observed [25]. It has been speculated that LAP-A may have a direct or indirect anti-nutritive role within the insect [27-29]. In contrast, LAP-A’s indirect role as a modulator of wound-signaling *in planta* is established [25]. LAP-A acts downstream of JA biosynthesis and perception to modulate the late branch of wound responses. After injury, *PPO-F*, *PinI*, and *PinII* RNAs accumulate to lower levels in *LapA-SI* relative to wild-type (WT) tomato plants. Reciprocally, these RNAs accumulate to higher levels and for extended times in *LapA-OX* plants. Early wound-response transcript levels are not influenced by changes in *LapA* RNA levels. 

 LAP-A is located within plastids and therefore must produce or control a signal (plastid → nucleus) to modulate tomato’s late wound-response genes [25,30]. Currently, the nature of the LAP-A-derived signal is unknown. LAP-A may modulate levels of a defense hormone that is synthesized within the plastid or a biogenic or operational retrograde signal. The role of biogenic retrograde signals in the regulation of photosynthesis-associated nuclear gene expression during light-regulated development and after disruption of plastid translation is well established [31-34]. Furthermore, operational retrograde signals critical for responses to biotic or abiotic stresses have been discovered including: ROS, redox signaling, PAP (3’-phosphoadenosine 5’phosphate), and MEcPP (methylerythritol 2,4-cyclodiphosphate)], as well as several dual-localized transcription factors [35-39]. A complete understanding of these operational signals has yet to be developed [31,32]. At this time, it is not clear if LAP-A’s well-studied peptidase activity and/or newly discovered molecular chaperone activity are critical for generating tomato’s retrograde wound signal [25,28,40].

To assess the potential for LAP-A having a broader role in defense/stress signaling and establish the tomato wound transcriptome, changes in tomato RNAs were determined in WT and *LapA-SI* tomato plants prior to and after wounding using cDNA microarrays. These analyses demonstrated that the tomato wound-response is complex, influencing the expression of a wide range genes associated with photosynthesis, as well as primary and secondary metabolism. Analysis of *LapA-SI* gene expression after wounding revealed that while overall gene regulation was similar to WT, *LapA-SI* responses were delayed after wounding. In addition, four new *LapA*-regulated genes (*PR-1c, PR-1a2*, *TAS14*, and *Dhn3*) were identified. Together this study demonstrates that LAP-A’s role in stress responses extends beyond the core late-wound signaling pathway. LAP-A serves as both a positive and a negative regulator of nuclear gene expression after injury.

## Results

### Functional annotation of wound-responsive DEGs: An overview

In order to identify transcriptome changes that occurred after injury in tomato, a cross-species hybridization (CSH) cDNA microarray study was used to assess RNA levels in wounded (local) and apical, non-wounded (systemic) leaves from WT plants at 0, 1 and 8 hr after wounding. Using the potato 10-K array (*Materials and Methods*) and a reference RNA design strategy [41], the spatial and temporal changes of RNAs in wounded leaves relative to non-injured leaves was determined. Differentially expressed genes (DEGs) were defined as up or down regulated based on a log_2_-fold change (|FC| ≥ 0.8) and a false discovery rate (FDR) of <5%. 

**Figure 1:**
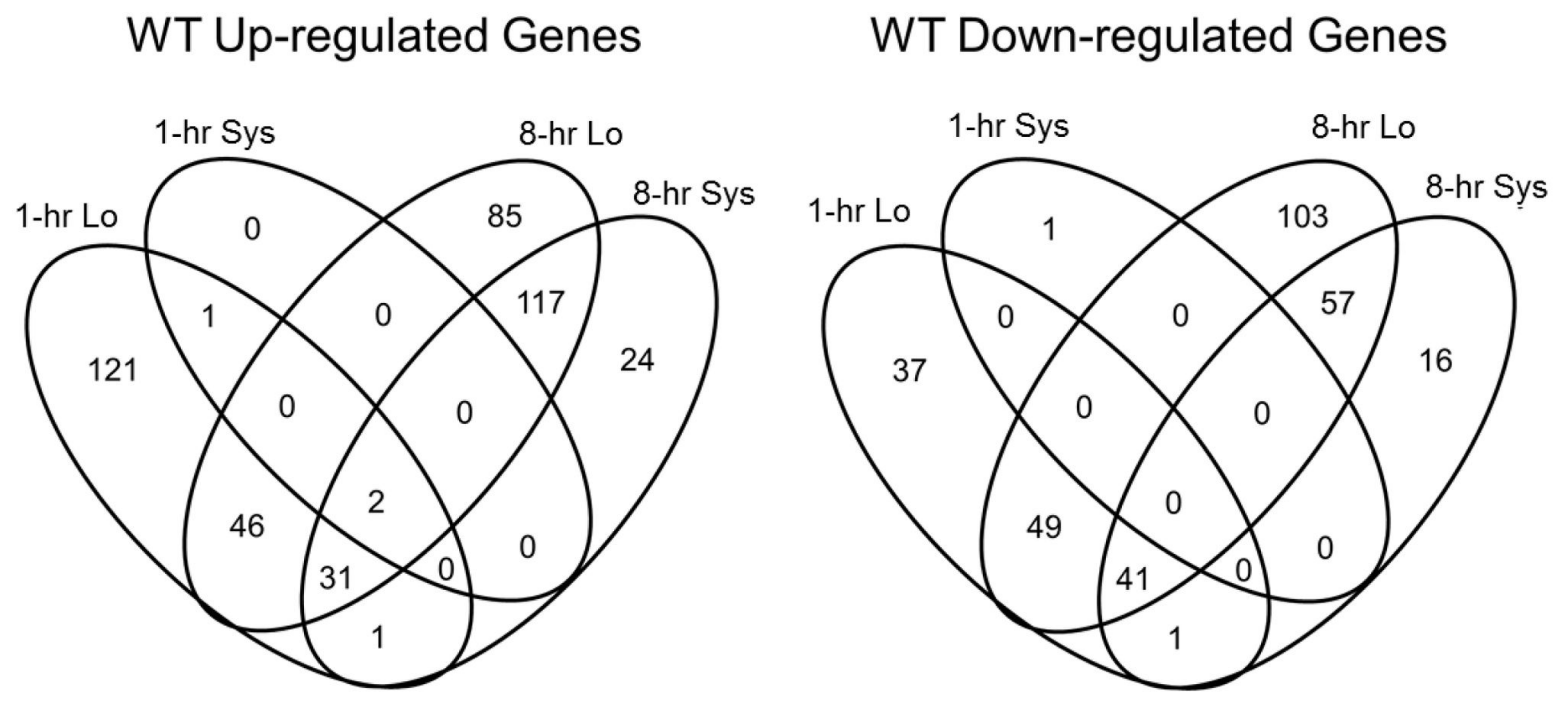
Gene expression patterns in WT plants at 1 and 8 hr after wounding. Genes that were differentially regulated in wounded (Local, Lo) and apical, non-wounded (Systemic, Sys) WT leaves at 1 and 8 hr after wounding were identified by analysis of the potato 10-K cDNA arrays (Materials and Methods). Differentially expressed genes (DEGs) were defined as those with *p* <0.05, |FC| ≥ 0.8.

 Changes in the tomato transcriptome after wounding were rapid. Within one hr after wounding, 330 DEGs were detected in the damaged leaves (Figure 1). Approximately 48% of the genes that responded at 1 hr (158 DEGs) were transiently expressed, with their RNA levels returning to pre-damage levels by 8 hr (Figure 1). Only four DEGs were detected in the systemic leaves at 1 hr after injury. These DEGs included three genes of unknown function, as well as the ethylene-responsive late embryogenesis protein ER5 [42] (Figure 1; Tables S1 and S2). 

 By 8 hr, there were 1.6- and 72-fold increases in the number of DEGs in damaged (531 DEGs) and undamaged, systemic (289 DEGs) leaves, respectively. Of the DEGs expressed in injured leaves, 362 of these RNAs were first detected at 8 hr (Figure 1). In addition, 83% the systemic DEGs were also detected in damaged leaves. Finally, a small number of 8-hr DEGs (24 up and 16 down) were unique to the apical, undamaged leaves suggesting that defense signaling in the systemic leaves may have unique features that are yet to be revealed (Figure 1; Table S1). 

 To investigate the types of genes responding to damage, DEGs were placed in 35 categories (BINs) based on their molecular function using MapMan [43-45]. DEGs with a wide variety of molecular functions were identified at both 1 hr and 8 hr after wounding (Table S1). While the number of DEGs substantially increased by 8 hr (Figure 1), the distribution of DEGs among the BINs at 1 and 8 hr after wounding were similar (Table S1). Statistical analysis was also performed using MapMan to test for enrichment of DEGs in any BINs (*Materials and Methods*). Five BINs (stress, photosynthesis, amino acid metabolism, tetrapyrole biosynthesis, and protein) and five subBINs (within the BINs for cell wall, RNA, lipid, and secondary and polyamine metabolism) were identified as differentially regulated after wounding (Table S1). Differentially regulated MapMan BINs/subBINs guided further dissection of the tomato response to wounding. Since only a small number of DEGs were identified in apical leaves at 1 hr (systemic DEGs) and since 83% of the 8-hr systemic DEGs overlapped with 8-hr local DEGs (Figure 1), detailed gene expression analysis was focused on the local wound response. A detailed analysis of wound-regulated DEGs appears in [46].

### Regulation of stress-responsive genes after wounding

 Consistent with previous studies, up-regulation of wound- and stress-responsive genes was a substantive component of the response to leaf injury (7% of total DEGs; Tables S1 and S3) [10,11,13,19]. Approximately 14% of the stress-related genes (BIN 20) on the array were differentially regulated after wounding. Of the 61 DEGs in this class, only 11 genes were down-regulated (Table S3). A majority of the down-regulated genes code for proteins associated with abiotic stress responses and have proposed functions in the regulation of water balance (putative major intrinsic proteins including aquaporins and tonoplast intrinsic proteins) and protein folding (homologues to HSP83, HSP80, HSP81-1, DnaJ) (Table S3). Down regulation of these water balance and chaperone genes was surprising given that breaches in tissue and cellular integrity can lead to dehydration and protein damage at the site of wounding [47]. The down-regulation of water channel protein genes may reflect the need to avoid an excessive water loss after injury. While counter-intuitive, the changes in chaperone gene expression may reflect the restoration of cellular homeostasis at the RNA level. 

 The majority of up-regulated DEGs in BIN 20 (stress) were spatially and temporally regulated (Table S3). For example, 14 of the 1-hr up-regulated DEGs were transiently induced (1-hr only). Several of these genes were associated with defense signal transduction including homologues of AtWRKY33, AtWRKY40, and AtWRKY75, a putative Leucine Rich-Repeat (LRR) receptor-like kinase, a whitefly-induced NAPDH oxidase (gp91-phox), and a Hin-1-like protein that has been associated with PAMP signaling (POTHR-1) [48-50]. It is possible that induction of these defense genes is important for limiting pathogen infection at the sites of tissue damage. Other genes solely expressed at 1-hr included a universal stress-related protein, stress-activated protein kinase and two Late Embryogenesis-Abundant (LEA) genes (*ER5* and *Dhn2*) (Table S3). The increases in *ER5* transcripts were consistent with previous studies that showed that *ER5* RNAs are rapidly and transiently induced by ethylene, drought, ABA, and wounding in tomato leaves [42]. 

 Other 1-hr stress-induced RNAs persisted until 8 hr and encoded proteins that may reduce the ability of pathogens to establish at the site of injury including: three pathogenesis-related (PR) proteins (PR-1c, PR-6, PR-10), three chitinases, and an endoglucanase inhibitor (Table S3) [51]. Other genes expressed at both 1 and 8 hr included several JA-induced proteins (JIPs) with established roles in defense (polygalacturonase, PPOs, and LAP-A) and additional peptidases (Ser carboxypeptidase and a cathepsin B-like proteinase). Cathepsin B is a member of the Cys proteinase superfamily, which includes enzymes that antagonize herbivores or modulate the hypersensitive response to plant pathogens [52,53]. To date, surprisingly little is known of cathepsin B’s role in wounding or insect defense. 

 A substantive number of stress-related genes (29 DEGs) were only detected at 8 hr after injury (Table S3). The 8-hr induced DEGs encoded additional JIPs, such as PPO-B, PinI, PinII, Cys protease inhibitors (cathepsin D inhibitor and cystatin), and a polygalacturonase inhibitor. Other well-studied JIPs such as threonine deaminase and arginase, with known anti-nutritive effects, were not present on the array [7]. 

 Surprisingly transcripts for several well-characterized genes encoding “early” wound-inducible response genes [*Lipoxygenase A* (*LoxA*), *Phospholipase A1*, and *Prosystemin*] were only detected at 8 hr after injury (Table S3) [15]. Previous studies have shown that *Prosystemin* and *LoxA* RNAs are detected in healthy leaves and increase by 0.5 hr to reach peak levels by 6 to 8 hr after wounding [15]. The absence of these RNAs at 1 hr may be due to differences in the wounding protocols, tomato genotypes or the inability of the CSH microarray to detect the small changes in tomato RNA levels [54,55]. The array data presented here provided the first evidence of tomato’s *phospholipase A1* RNAs increasing after injury, which correlates with the rapid increase phospholipase A activity after wounding in tomato [56] and is consistent with increases in *Arabidopsis Phospholipase A* RNAs after wounding and abiotic stress [57]. 

### Regulation of photosynthesis and reactive oxygen species metabolism

 Although some exceptions are known, the global down-regulation of photosynthesis gene expression appears to be a response that has been subject to evolutionary selection, since it occurs in many plant-pathogen and -herbivore interactions [9,58]. Consistent with these findings, 10-12% of the down-regulated DEGs at 1 and 8 hr after wounding encoded proteins for tetrapyrole metabolism (BIN 19) and photosynthesis/carbon fixation (BIN 1), including PSI, PSII, light harvesting complexes, and the blue-light receptor phototropin 2 (Tables S1, S5, and S6). While these changes were most dramatic in the injured leaves, the decline in some BIN 1 and BIN 19 RNAs was also observed systemically. 

 While H_2_O_2_ and other ROS are essential for wound signaling in tomato [59], ROS are toxic at higher concentrations and must quickly be catabolized. For this reason, it was not surprising that 13% of the ROS metabolism genes (BIN21) on the array were DEGs (Table S7). However, counter-intuitively, almost half of DEGs encoding ROS catabolism proteins were down-regulated after injury, suggesting there is a complex regulation of ROS metabolism during the tomato wound response, perhaps involving temporal and/or spatial regulation of different ROS species. Interestingly, a majority of the down-regulated ROS metabolism genes encoded proteins localized to the plastid and none of the up-regulated ROS gene products were plastid localized. This is consistent with previous studies that showed that the localization rather than function, determined regulation of ROS metabolism genes after stress [9]. The reduced levels of ROS catabolism RNAs may reflect the need for plastid-generated ROS, which can serve as an anti-microbial agent or a retrograde signal to enhance or modify wound responses [31,58,60].

### Regulate the regulators: RNA and protein metabolism

 The array monitored a large number of genes involved in regulating gene expression at a variety of levels from transcriptional to post-translational control [BIN 27 (RNA) and BIN29 (Protein), respectively]. Approximately 14% of the DEGs at 1 and 8 hr after wounding were involved in RNA metabolism (BIN 27), which included 801 transcription factors and their accessory proteins, as well as RNA-binding proteins (Tables S1 and S6). Evidence for early and late phases of transcription factor RNA accumulation was noted with 26 “early” (1-hr only), 27 “late” (8-hr only), and 8 “persistent” (1-hr and 8-hr) DEGs (Tables S4 and S6). 

 A substantive number of genes (56 genes) involved with protein metabolism (BIN 29: protein synthesis, modification, degradation, or sorting) were regulated after injury (Tables S4 and S6). Consistent with the preferential decline in RNAs encoding proteins destined for the chloroplast, several plastid ribosomal protein RNAs were down-regulated DEGs. In addition, wounding impacted genes in the protein folding and post-translational modifications BINs. For example, nine DEGs encoded kinases and phosphatases that may control the phosphorylation status of proteins involved with transcriptional cascades, ER to nucleus signaling (IRE1-like protein gene), and/or protein turnover [61,62]. 

 The majority of protein metabolism DEGs were involved in protein degradation. With a small number of exceptions, these DEGs were up-regulated and doubled in number by 8 hr (Tables S4 and S6). These DEGs included polyubiquitin, a putative F-box family member, LAP, Cys and Asp proteinases, and Ser carboxypeptidases. Proteinases may have multiple roles in the wound response including generation/catabolism of bioactive peptides or retrograde signals to control defense signaling [25,63,64] or directly interfering with insect midgut integrity or nutritional value of food [65,66]. In addition, these enzymes may be essential for the re-establishment of cellular homeostasis in cells surrounding the wound site by degrading damaged proteins to prevent the accumulation of unfolded proteins, which can aggregate and induce cell death, and providing a pool of amino acids for cellular recovery [65]. 

### Strengthening cell walls

 Consistent with the need to fortify physical barriers against opportunistic pathogens [47,67], a predominant response of tomato leaves after wounding was activation of cell wall-strengthening genes; 12% of the DEGs resided in the cell wall or secondary metabolism BINs (BIN 10 and 16, respectively; Table S7). Of particular importance are the genes involved in phenylpropanoid biosynthesis. These metabolites reinforce the cell wall serving as cell-wall bound phenolics, lignins, suberin, and cuticle-associated phenolics [68]. In addition, phenylpropanoids can be oxidized by wound-induced PPOs to form toxic quinones that have anti-feedant and toxic effects on insects [69]. 

**Figure 2:**
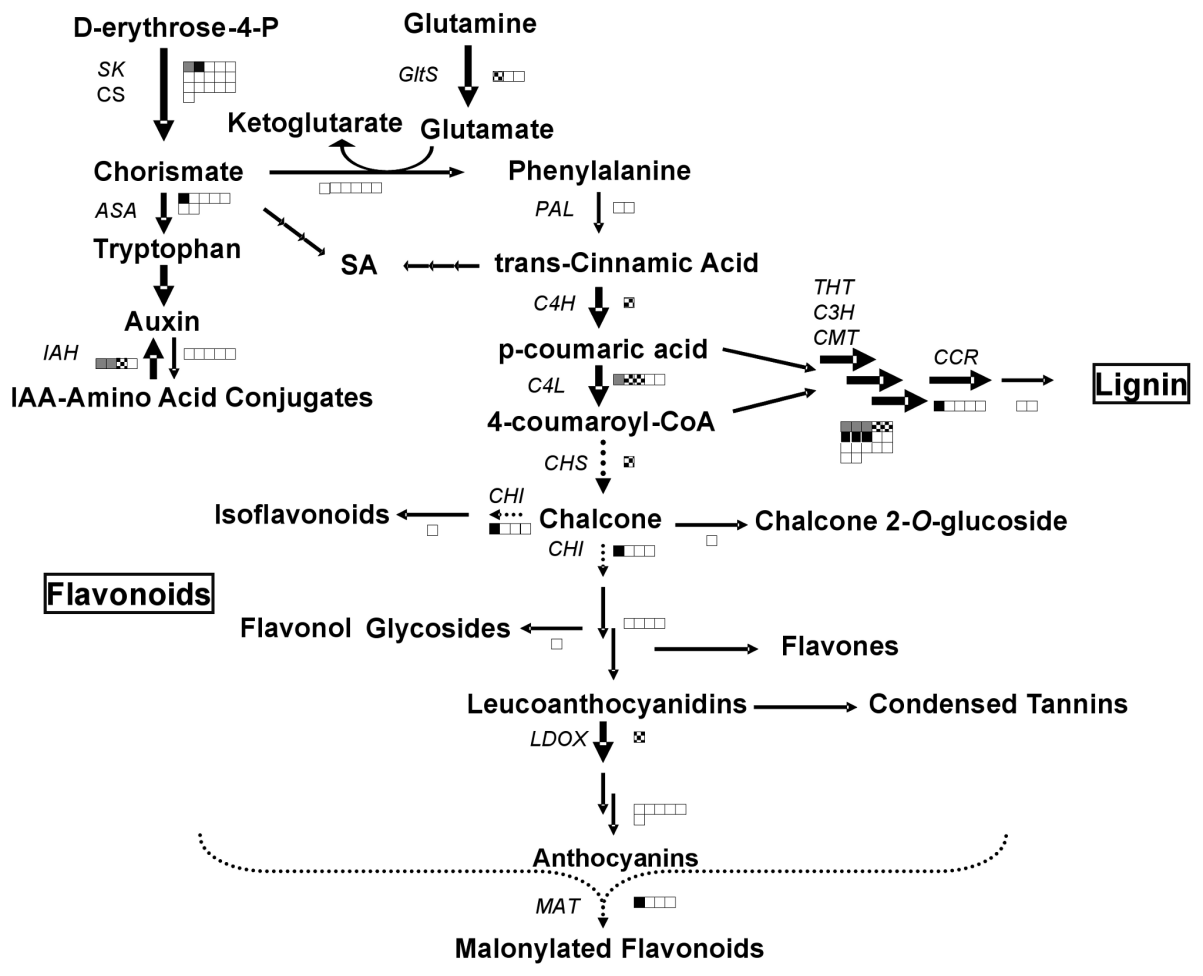
Changes in phenylpropanoid synthesis and catabolism gene RNAs after wounding. Many genes involved in phenylpropanoid metabolism were wound-regulated DEGs. For each biochemical step the number of genes regulated out of total number of clones representing those genes on the TIGR 10-K (version 3) potato cDNA microarray is indicated as a single or cluster of blocks. The colored blocks represent DEGs regulated at 1 hr (grey) and 8 hr (black) or both (checkered). Up-regulated DEGs are indicated by solid arrows and down-regulated DEGs are represented by dotted arrows. For some biochemical steps, enzymes were not represented on the array; no boxes appear at these steps. For complete pathway see Plant Metabolic Network (PMN; pmn.plantcyc.org). *PAL, phenylalanine* *ammonia-lyase; C4H, cinnamic* *acid 4-hydroxylase; C4L, 4-coumarate-CoA* *ligase; CCR, cinnamoyl-CoA* *reductase; CHI, chalcone* *isomerase; LDOX, Leucoanthocyanidin dioxygenase; CHS, Chalcone synthase; MAT, Malonyltransferase; ASA, Anthranilate* *synthase* *alpha; SK, Shikimate kinase; CS, Chorismate synthase; GltS, Glutamate synthase; THT, Tyramine* *N-hydroxycinnamoyl* *transferase; C3H, P-coumaroyl* *shikimate 3'-hydroxylase; CMT, Caffeoyl-CoA* *O-methyltransferase*.

 After wounding, most tomato RNAs encoding enzymes for phenylpropanoid biosynthesis (BIN 16) increase. In this study, the exception is the gene that encodes phenylalanine ammonia lyase (PAL), the first and committed step to phenylpropanoid biosynthesis. While the tomato *PAL5* RNA is known to increase in response to wounding, JA, ethylene and ABA [73,74], *PAL* was not detected as a DEG in this study (Table S2; Figure 2). This is likely due to the facts that *PAL5* RNAs are present at high levels in healthy tomato leaves and current tomato cDNA sequences indicate that several additional *PAL* gene transcripts may cross-hybridize with the potato PAL spots on the array. In contrast, our study does show that many of the other RNAs for core phenylpropanoid biosynthetic genes such as *Cinnamic Acid 4-Hydroxylase* (*C4H*) and *4-Coumarate:Coenzyme A Ligase* (*C4L*) are DEGs (Figure 2; Table S7). The C4H and C4L products are used for synthesis of lignins (monolignols) or anthocyanins (flavonoids) [68]. After injury in tomato leaves, monolignol biosynthesis gene RNAs increased; in contrast, the rate-limiting flavonoid biosynthetic gene (*Chalcone Synthase*; *CHS*) RNAs declined (Table S7; Figure 2). Furthermore, there was a down-regulation of cell wall-degrading and -remodeling enzyme RNAs (BIN 10) at both 1 hr and 8 hr after wounding (Table S7) [67]. Collectively, these data support the premise that there is increased lignification and fortification of the cell wall at the expense of cell wall flexibility/expansion and production of flavonoids after wounding in tomato (Figure 2). 

### Lipid and jasmonate metabolism and signaling

 The roles of lipids and jasmonates in defense and wound signaling are well established and evidence for differential regulation of lipid metabolism genes (BIN 11) after injury of tomato leaves was found using the CSH arrays [6,70]. Fatty acid desaturases (FADs; BIN 11.2) catalyze the formation of double bonds in the lipid tails of fatty acids. The wound-induced tomato *FAD7*, also known as *SPR2* (*Suppressor of Prosystemin mediated responses2*), provides precursors for defense-related oxylipins and JA [71]. Other tomato FADs are less well characterized and only the *FAD5, FAD8* and *FAD2* genes were represented on this array. This microarray provided the first report that injury causes a decline in the RNAs encoding the plastid-localized FAD5 and FAD6 (Table S7). If FAD5/6 activity also declines in parallel with *FAD5/6* RNAs, this may be important mechanism for restoring JA and other lipid levels to non-stress levels. FAD2 is located in the ER and is involved in defense-independent lipid metabolism [70]. In addition*, FAD2* RNA levels increase in response to insect oral secretions in potato [20] and JA treatments and whitefly feeding in *Arabidopsis* [11,72,73]. The CSH microarray data presented here provided the first evidence that the two tomato *FAD2* genes were reciprocally and temporally regulated with an increase in *FAD2.1-like* RNAs (1 hr) and a decline in *FAD2.2-like* RNAs (8 hr) after wounding (Table S7). 

 LOXs catalyze the addition of oxygen to linoleic or linolenic acids and have a key role in defense and wound signaling. 13-LOX-derived lipids are precursors for JA [6] and while the role for 9-LOX-derived oxylipins is less well-characterized, they have also been implicated in defense and mitochondria-to-nucleus retrograde signaling [74-76]. The array monitored one *13-LOX* gene (*LoxC*), one *9-LOX* gene (*LoxA*) and a *Lox6* gene that has not been characterized to date in tomato. Increases in only *LoxA* and *Lox6* RNAs were detected at 8 hr after injury (Table S3). Many wound-responsive genes associated with JA and JA-Ile biosynthesis were not on the array including: *LoxB* (a 9-LOX gene), *LoxD* (a 13-LOX gene)*, Allene Oxide Synthase*, *Allene Oxide Cyclase*, *Acyl-CoA Oxidase*, *12-OPDA reductase* 3, as well as *Jasmonic acid responsive 1* [6,18,19,77]. 

 Linolenic acid is also used to produce six-carbon, green leaf volatiles (GLV), which have both direct and indirect roles in plant-herbivore interactions [78] and divinyl ether fatty acids, which have been implicated in pathogen defense [70]. The array monitored expression of two GLV biosynthesis genes encoding the rate-limiting hydroperoxide lyase (HPL; BIN 20) and alcohol dehydrogenase (ADH2; BIN 5) and one divinyl ether synthase (DES; BIN 17.7). These RNAs were not injury induced (Table S2), which is consistent with previous studies in the Solanaceae [17,19,79] but contrasts with *Arabidopsis* [11,78]. 

### Defense modulators - ET and SA

**Figure 3:**
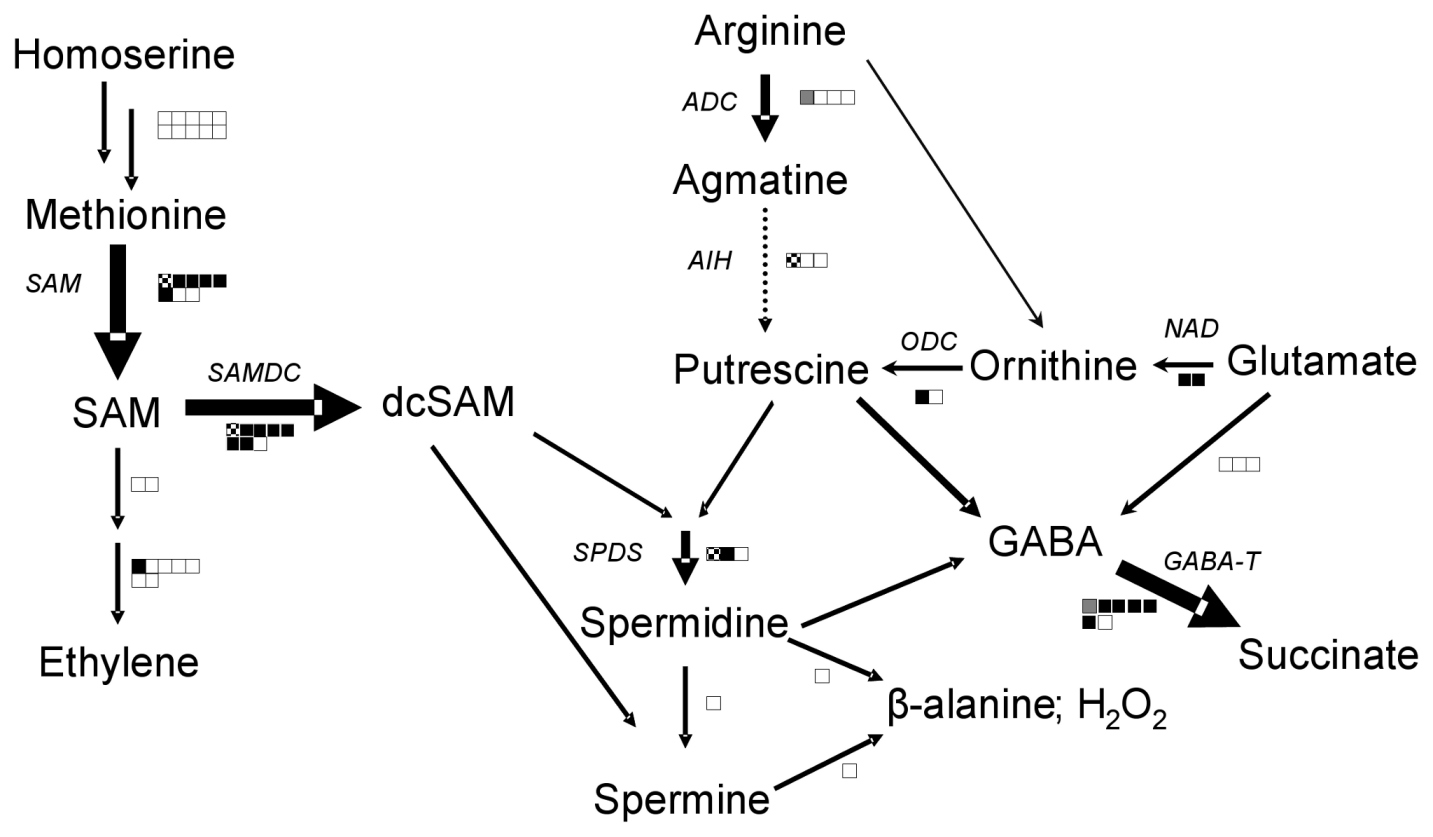
Changes in polyamine, SAM, and ethylene synthesis and catabolism gene RNAs after wounding. Many genes involved in polyamine, SAM, and ethylene metabolism were wound-induced DEGs in tomato. DEG expression patterns are represented as described in Figure 2. *SAM, S-adenosylmethionine* *synthetase; SAMDC, S-adenosylmethionine* *decarboxylase; SPDS, Spermidine synthase; ADC, Arginine* *decarboxylase; AIH, Agmatine* *iminohydrolase; ODC, Ornithine* *decarboxylase; NAD, N-acetylornithine* *deacetylase; GABA-T, GABA* *Transaminase*.

 Ethylene’s (ET) role in defense signaling is species dependent [80,81]. In tomato, ET treatments increase *PR* gene RNAs but not the canonical wound-response *PinII* gene RNAs [82,83]. However, studies with an ET-perception mutant and pharmacological studies show that ET is essential for a robust wound response in tomato [84]. The microarray data presented here is consistent with a modest ET injury response with up-regulation of only four genes encoding aminocyclopropane-1-carboxylate oxidase (ACO), ER5, and two ET-responsive transcription factors similar to ABR1 and RAP2.7 (Tables S6 and S8, Figure 3) [42,85]. 

 Salicylic acid (SA) is a key defense hormone involved in the cross-talk of defense networks [12,81]. There was a paucity of SA biosynthesis/modification and SA perception genes on the 10-K array (BIN 17.8; Tables S2 and S8) [86]. Of the three SA biosynthesis/modification RNAs monitored, none changed in response to wounding (Figure 2; Table S2). The key genes involved with SA perception (*NPR3, NPR4*) and signaling transduction (*NPR1*) were not present on the potato array. However, the array showed that the NPR1-interacting protein *TGA2* RNAs increased in damaged leaves by 8 hr (Table S8) [87]. TGA2 controls SA-mediated defenses and innate immunity in tomato and presumably enhances defenses to protect against opportunistic pathogens after injury (Table S8).

### Isoprenoid hormones and wounding

 Isopentenyl diphosphate (IPP) is the building block for the synthesis of the large and functionally and structurally diverse set of metabolites called isoprenoids [88]. IPP is synthesized in the plastid using the 2-C-methyl-D-erythritol 4-phosphate (MEP) pathway leading to production of monoterpenes, antioxidant carotenoids, and photosynthetic metabolites, as well as ABA, cytokinins (CK), and gibberellic acid (GA). The mevalonate (MVA) pathway produces IPP for cytoplasmic or mitochondrial isoprenoids including sesquiterpenes, sterols and brassinosteroids (BRs). 

 The microarray analyses indicated that none of the core MVA- or MEP-pathway genes were DEGs (BIN16; Table S2) and the majority of sesquiterpenoid and monoterpene biosynthesis genes on the array were not DEGs (Table S7). The lack of regulation of these genes at the RNA level was surprising, since levels both monoterpenes and sesquiterpenes increase after wounding/herbivory in tomato [89,90] and RNAs encoding the rate-limiting enzymes for the MVA and MEP pathways (*3-hydroxy-3- methylglutaryl-coenzyme A reductase and 1-deoxy-D-xylulose 5-phosphate synthase*, respectively) increase in response to MeJA and wounding in potato and herbivory in tomato [90-92]. These data suggest that regulation at other levels of gene expression occurs in the tomato cultivar used for this study, as has been noted in other species [88], or the CSH array was not able to detect small changes in the levels of these transcripts. 

 The ability to perceive MEP pathway-derived CK is important for SA and JA accumulation after wounding in tobacco [93]. However, in tomato, none of the 14 genes involved in CK biosynthesis or signaling/response were DEGs (BIN 17.4; Table S2). This suggests that CK’s role after injury may be different in tomato and tobacco, regulation must occur at a different level of gene expression, or the changes in CK biosynthesis/response gene RNAs are small, thereby evading CSH detection.

 ABA levels increase after injury and are essential for a robust wound response in tomato [94]. Only two genes dedicated to ABA biosynthesis were present on the array (BIN17.1; Table S2). *NCED1* (*9-cis-Epoxy-Carotenoid Dioxygenase 1*) and *NCED4* encode the rate limiting enzymes for ABA biosynthesis [95]. Only *NCED1* was a DEG with its RNAs increasing in damaged leaves early (Table S8). 

 In tomato, GA antagonizes wound signaling [96]. Of the 17 genes involved in GA biosynthesis, signaling or responses (BIN 17.6; Tables S2 and S8), only two were DEGs. *Ent-Kaeurenoic Acid Oxidase* (*KAO*) RNAs increased locally and systemically. KAO synthesizes GA_12_, which is a precursor to the bioactive GA forms (GA_1_, GA_3_, and GA_4_) [97]. While regulation of GA synthesis is known to occur at the transcript level in other plants, *GA oxidase* rather than *KAO* transcripts are typically regulated [97]. The *GA-regulated* Transcript *1* (*GAST1*) RNAs decreased after leaf injury. *GAST1* transcription is known to be induced by GA and suppressed by ABA in tomato leaves [98]. In addition, *GAST1* RNAs increase in response to herbivory in potato and tomato [20,99] suggesting that herbivore elicitors may induce *GAST1* gene expression suggesting unanticipated complexities of GA and herbivory. 

 Finally, BR is derived from cytosolic isoprenoids and is a negative regulator of JA-regulated defenses in tomato [100]. Several sterol and BR biosynthetic genes were DEGs (Bin 17.3; Table S7). *Squalene monooxygenase/epoxidase* RNAs decreased 8 hr after wounding (Table S8). In contrast, RNAs for a gene encoding a rating-limiting enzyme (C-8,7 sterol isomerase) and several BR biosynthesis enzymes [DWARF1, DWARF1-like, steroid 5-α-reductase (DET2)] increased after injury (Table S8). The microarray data suggest that BR responses may be complexly regulated, for none of the 11 BR-response/signaling genes were DEGs.

### Amino acid-derived signaling molecules in defense (IAA, polyamines, GABA)

 MapMan identified the amino acid metabolism BIN as differentially regulated after wounding (BIN 13; Tables S1 and S7). Amino acid pools are critical for synthesis of proteins, auxin, polyamines, and γ-aminobutyrate (GABA). Trp is a precursor to auxin and indole alkaloids [101]. The first committed step to Trp biosynthesis is catalyzed by anthranilate synthase (AS). While *AS1* RNAs increased after injury (Table S7), other auxin biosynthetic genes and the five *IAA-amido synthetase* genes (responsible for forming IAA-amino acid conjugates) were not DEGs (BIN 17.2; Figure 2; Tables S2 and S8) [102]. In contrast, three *IAA-amino acid hydrolase* RNAs accumulated in damaged leaves (Table S8); if the changes in *IAA-amino acid hydrolase* RNAs are paralleled with increases in hydrolase activities, there may be more active IAA, which is known to enhance JA signaling in tomato roots [103]. In addition, two negative regulators of auxin signaling (*AUX/IAA* genes: *IAA2.3-like* and *IAA28-like*) were down-regulated late after injury, which was also consistent with this theory (Table S8) [104]. 

 One of the most prominent changes after injury was up-regulation of polyamine biosynthesis genes (BIN 22; Table S7). Polyamines are polycationic compounds with roles in abiotic/biotic stress [105]. Over 50% of the polyamine biosynthesis genes on the array were up-regulated after wounding including genes encoding arginine decarboxylase, ornithine decarboxylase, agmatine iminohydrolase, and N-acetylornithine deacetylase (Table S8; Figure 3). In particular, RNAs encoding spermidine synthase and enzymes critical for decarboxylated S-adenosylmethionine (dcSAM) biosynthesis (SAM synthetase and SAM decarboxylase) increased markedly after wounding (BIN 22.1.2; Figure 3; Table S8). This is consistent with the fact that SAM decarboxylase transcript levels are correlated with polyamine levels and polyamines and polyamine biosynthesis RNAs increase after MeJA treatments and pathogen infection in tomato [106,107]. The oxidation of polyamines generates H_2_O_2_, which strengthens cell walls and serves as a mobile defense signaling molecule; polyamines are also used to generate toxic phenylpropanoid-polyamine conjugates (PPC) [108,109]. PPCs slow insect growth, are antimicrobial and strengthen the plant cell wall [108,110]. Since the phenylpropanoid pathway is also up-regulated after wounding (discussed above), the wound-induced polyamines are likely to be channeled in part toward PPC production.

 The Glu and the polyamine pathways also produce GABA (Figure 3) [111]. GABA antagonizes insect growth and increases resistance to herbivory in several plant species. GABA levels rapidly increase in response to wounding, insect crawling and herbivore feeding through post-translational regulation of glutamate decarboxylase (GAD) [111,112]. While genes for enzymes that convert putrescine and spermidine to GABA were not present on the array, *GAD* RNAs did not increase in response to mechanical wounding (BIN 13; Table S2). Interestingly, several RNAs encoding GABA transaminase, which catabolizes GABA, increased after wounding suggesting that wound-induced increases in GABA may be rapidly depleted to restore homeostasis (Figure 3; Table S7).

### LapA-SI has delayed responses after wounding

 LAP-A is critical for mounting an effective defense against chewing insects [25]. LAP-A controls the abundance and persistence of late wound-response RNAs, such as *PinI*, *PinII*, and *PPO-F*, but does not influence early wound-response RNAs [25]. To broaden our understanding of the scope of LAP-A’s impact on tomato’s gene expression programs, the local and systemic injury responses in WT and *LapA-SI* plants were compared at 0, 1, and 8 hr after wounding using the potato cDNA arrays. RNA levels relative to the WT 0-hr control (FC) were determined and statistical analysis was performed as before. 

**Figure 4:**
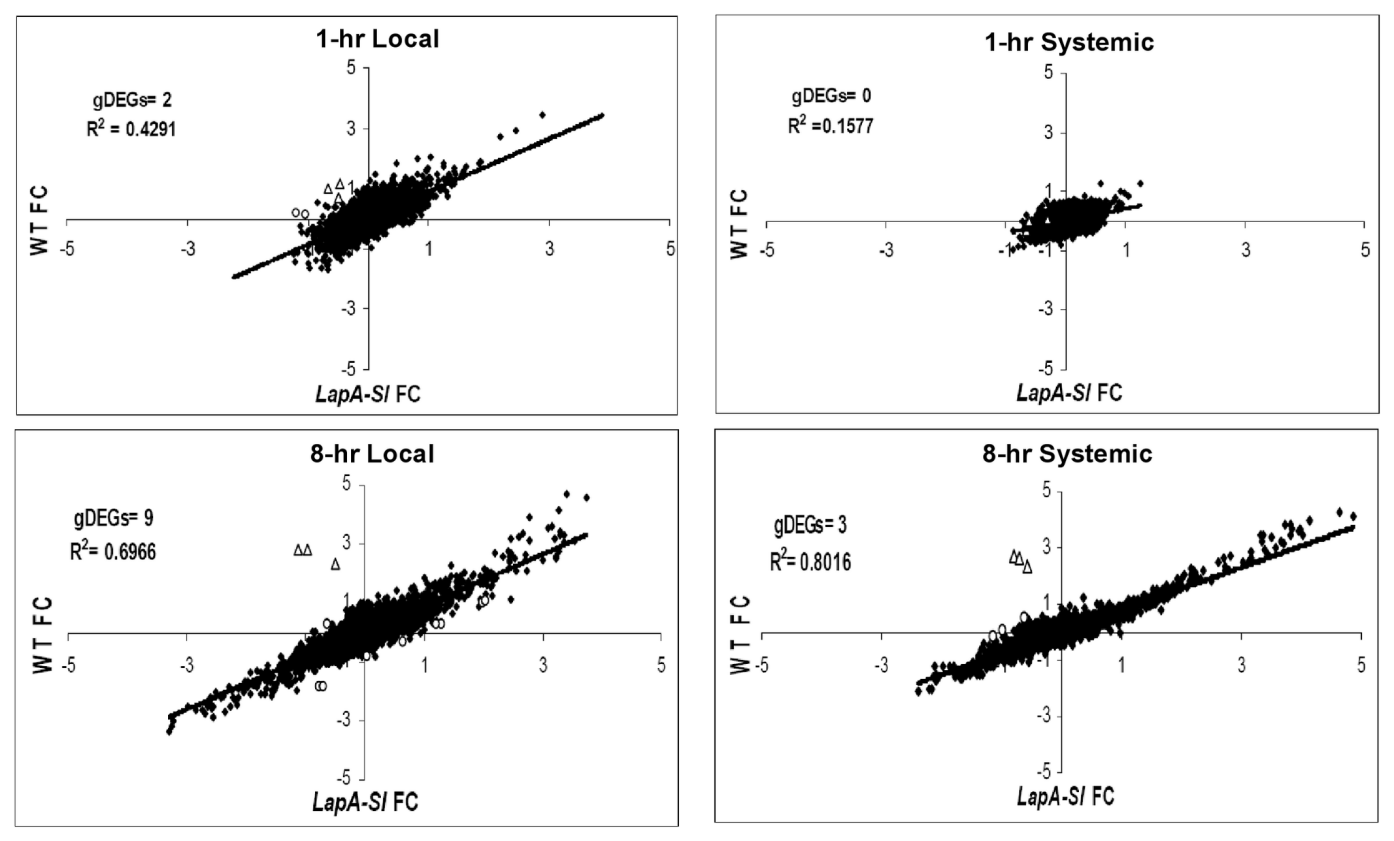
Relative RNA fold change in WT vs. ***LapA-SI* lines 1 and 8h after wounding**. Correlation of relative RNA fold change (FC) between WT and *LapA-SI* lines after wounding. RNAs accumulated to similar levels in WT and LapA-SI leaves are indicated by closed diamonds. The genes differentially regulated between in *LapA-SI* and WT leaves are indicated by open circles (genotype DEGS (gDEGs); p<0.05, |FC| ≥0.8]). *LapA* RNAs are indicated by open triangles.

 The local and systemic gene expression trends at 1 hr and 8 hr after injury in WT versus *LapA-SI* leaves are displayed in Figure 4. Overall gene expression was correlated between the genotypes in all samples. The strongest correlation between WT and *LapA-SI* DEGs was in systemic leaves at 8-hr after wounding (R^2^= 0.8016). Only a few genes were differentially regulated (p<0.05, |FC| ≥0.8) in the *LapA-SI* line after wounding relative to WT plants (genotype DEGs or gDEGs). As expected, *Lap* transcripts were absent in the *LapA-SI* line (Figure 4; Table 1). Surprisingly, none of the previously characterized wound-responsive genes (*PinI, PinII* and *PPO*) that were strongly *LapA* dependent in previous studies were statistically different in the *LapA-SI* lines at 1 and 8 hr after wounding. This is likely due to the fact that LAP-A had largest impacts on transcript abundance at 12 to 24 hr after wounding [25]. 

**Table 1:** Genotype DEGs at 1 and 8 hr after wounding^A^.

				**WT**					***LapA-SI***				
				**0 hr**	**1 hr**		**8 hr**		**0 hr**	**1 hr**		**8 hr**	
**Time Point**	**BIN**	**Clone ID^B^, ^C^**	**Annotation**		**Local**	**Systemic**	**Local**	**Systemic**		**Local**	**Systemic**	**Local**	**Systemic**
1-hr Local	34	STMDJ91*	Similar to vacuolar proton pump	0.04	0.17	0.00	0.28	0.16	-0.81	-1.19	-0.72	-0.63	-0.66
	34	STMFB95*	Homologue to chloride channel protein	0.11	0.11	0.12	0.15	0.03	-0.70	-1.03	-0.44	-0.50	-0.76
8-hr Local	20	STMGZ34^†^	Heat shock cognate protein 80	-0.11	-0.14	-0.01	-0.72	-0.37	-0.05	0.12	0.07	-0.05	-0.30
	20	STMIB24*	Similar to DnaJ-like protein	-0.22	-0.32	-0.41	0.29	-0.35	-0.02	-0.83	-0.47	-0.63	-0.91
	29	STMIX54^†^	Similar to ATP-dependent Clp proteolytic subunit	-0.04	0.23	-0.10	0.30	0.02	0.10	0.64	0.32	1.20	0.25
	29	STMIX83^†^	Similar to 60S ribosomal protein L6	-0.23	-0.48	-0.13	-0.79	-0.28	-0.62	-0.22	0.01	0.04	0.00
	20/29	STMEU11^†^	Homologue to cathepsin B cysteine proteinase	-0.01	0.76	-0.14	1.05	0.64	-0.37	0.61	0.21	2.05	1.20
	10.2	STMCZ18*	endo-1,4-beta-glucanase	0.17	0.19	0.23	0.23	0.02	0.49	0.00	0.24	-0.39	-0.08
	34	STMCC78^B†^	Similar to ABC transporter F family protein 5	-0.39	-0.61	-0.60	-1.86	-1.55	-0.53	-0.16	-0.19	-0.69	-1.59
	25	STMEA95^†^	Weakly similar to carboxylesterase	-0.16	0.19	-0.12	0.29	-0.10	0.17	0.40	0.05	1.30	0.51
	26	STMHX28^†^	Similar to UDP-glycosyltransferase	-0.20	-0.18	-0.36	-0.31	-0.32	0.06	-0.06	-0.36	0.64	-0.06
8-hr Systemic	10.7	STMHG34*	Xyloglycan endo-transglycosylase	0.06	-0.01	-0.38	-0.19	0.48	0.12	0.02	-0.45	-0.59	-0.61
	35	STMGV86*	Similar to hypothetical protein VITISV_011279*	0.05	0.09	-0.16	0.05	0.04	0.20	-0.75	-0.37	-0.82	-0.99
8-hr Local & Systemic	29	STMCB75*	Similar to 50S ribosomal protein L1	-0.13	-0.47	-0.10	-0.53	-0.20	-0.64	-1.01	-0.41	-1.27	-1.15

^A^ At each experimental point, the |FC| ≥0.8 (p<0.05) of each gene was determined. gDEGs were identified by comparing gene signals in the WT and *LapA-SI* lines.

^B^ gDEGs that have RNAs at lower levels in *LapA-SI* relative to WT are indicated with an asterisk. gDEGs that have RNAs at higher levels in *LapA-SI* relative to WT are indicated with a ^†^

^C^ Two cDNAs (STMCC78 and STMDP40) representing the ABC transporter F family protein 5 gene were on the TIGR 10-K (version 3) microarray. Signals from STMCC78 are shown.

 Fourteen other genes were identified as gDEGs at 1 and/or 8 hr after wounding (Figure 4; Table 1). Two genes impacting ion transport (a vacuolar proton pump and chloride channel protein) were suppressed 1 hr after injury in *LapA-SI* leaves relative to WT leaves. By 8 hr, many of the gDEGs encoded proteins involved in stress (BIN 20) and/or protein metabolism (BIN 29) including HSP80, a DnaJ-like protein, ClpP, and cathepsin B. Seven of the ten gDEGs identified at 8-hr in injured leaves were up-regulated in *LapA-SI* relative to WT. These data indicated that LAP-A’s impact on responses to injury is broader than previously recognized [25].

 In addition to identifying a small number of gDEGs after injury, the overall responsiveness of the *LapA-SI* lines to wounding was delayed relative to WT plants. For example, the number of DEGs (FC| ≥ 0.8) identified in *LapA-SI* leaves 1 hr after wounding (149 DEGs) was less than half of those in the WT plants (329 DEGs) (Table S1). In addition, there was a trend of lower expression of stress-related DEGs (BIN20) in the *LapA-SI* lines compared to WT (Table S3); this was most striking at 1 hr. Therefore, to identify the genes that were differentially regulated in the *LapA-SI* plants at 1 hr, the top 100 genes with the largest fold differences (|FC|=0.72-1.66) between *LapA-SI* and WT in 1-hr damaged leaves were compared (Table S9). A majority of these genes were not identified as significantly different due to high variation associated with CSH comparisons. However, these analyses showed a compelling trend. A majority of these RNAs (78%) were at lower levels in the *LapA-SI* relative to WT leaves. Interestingly, when the top 30 most suppressed genes were viewed, 12 of the cDNAs encoded proteins involved in stress responses (BIN 20). No enrichment of BINs was seen in the 22 genes with an up-regulation trend in *LapA-SI* plants (Table S9).

 By 8 hr after wounding, the numbers of local and systemic DEGs in *LapA-SI* vs WT plants were similar (Table S1). However, inspection of the top 100 most differentially expressed genes at 8 hr after injury (|FC|=0.72-3.93) in the *LapA-SI* vs WT plants indicated that the majority of genes (74%) had lower RNA levels in *LapA-SI* than WT (Table S10). Different BINs were preferentially represented in the top 30 suppressed and induced genes in *LapA-SI* plants. By 8 hr, only six of the top 30 suppressed genes were stress-related (BIN 20) and ten of the 26 up-regulated genes were associated with protein metabolism (BIN 29). These genes encoded proteases (ClpP, cathepsin B), abiotic stress-response proteins (HSP90, TAS14) and PR proteins (PR-1b, class II chitinase, and endo-1,3-β-glucosidase). While subject to variability, together the 1-hr and 8-hr trends suggest that LAP-A modulation of stress responses was more complex and occurred earlier after injury than previously realized.

### LAP-A impacts gene expression of PR-1 and late wound-induced dehydrins

**Figure 5:**
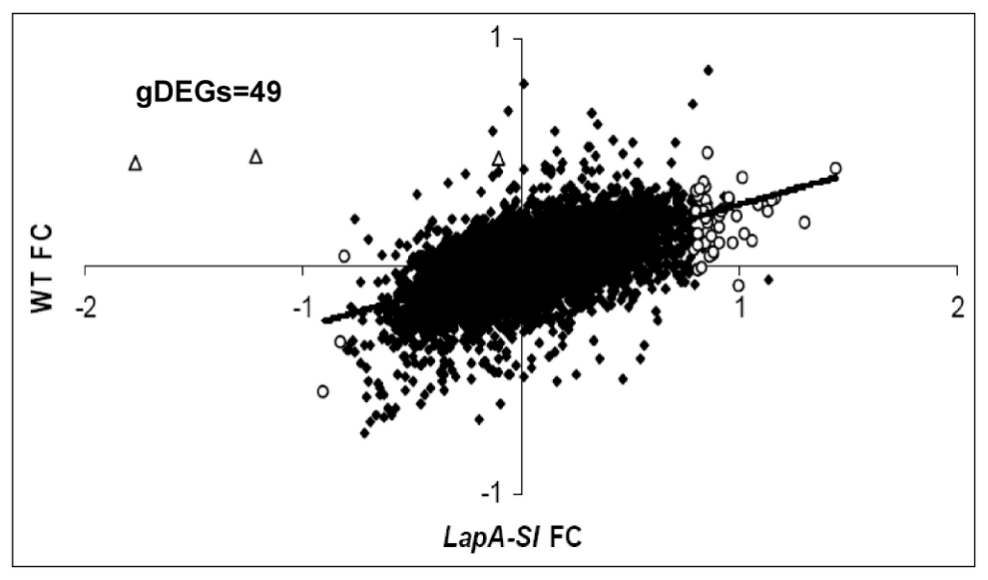
Genes differentially regulated in *LapA-SI* before wounding (putative 0-hr gDEGs). Genes with RNAs at similar levels in *LapA-SI* and WT leaves before wounding (0 hr) are indicated with closed diamonds (log_2_ fold change (FC) <1.5). Putative 0-hr g*DEG*s are indicated with open circles (p<0.05, |FC| ≥0.8). *LapA* RNAs are indicated by open triangles.

 While a small number of DEGs were identified in the *LapA-SI* line compared to WT after mechanical wounding (Table 1), there was a set of 49 genes that were predicted to be differentially regulated by LAP-A before injury (0-hr gDEGs) (Figure 5; Tables S11 and S12). A majority of the putative 0-hr gDEGs were up-regulated in the *LapA-SI* line and were co-regulated 1 hr and 8 hr after wounding (Figure S1; Table S11). However, when homologs of seven potato ESTs were identified and their RNA levels were monitored by RT-PCR or qPCR (Tables S11 and S12; *Materials and Methods*), no 0-hr gDEGs could be confirmed. This could be due to the complications of utilizing CSH arrays as discussed below including the fact that many of the potato ESTs examined had high sequence identity with more than one tomato gene (Table S12). In addition, the RT- and qPCR data indicated that four of the genes characterized (*SlWRKY42, MYBR29-like, VACULOLAR ATPase*, and *BEL1-like*) encoded rare class RNAs and therefore differences in RNA levels may have been obscured by biological variation between replicates (data not shown). 

**Figure 6:**
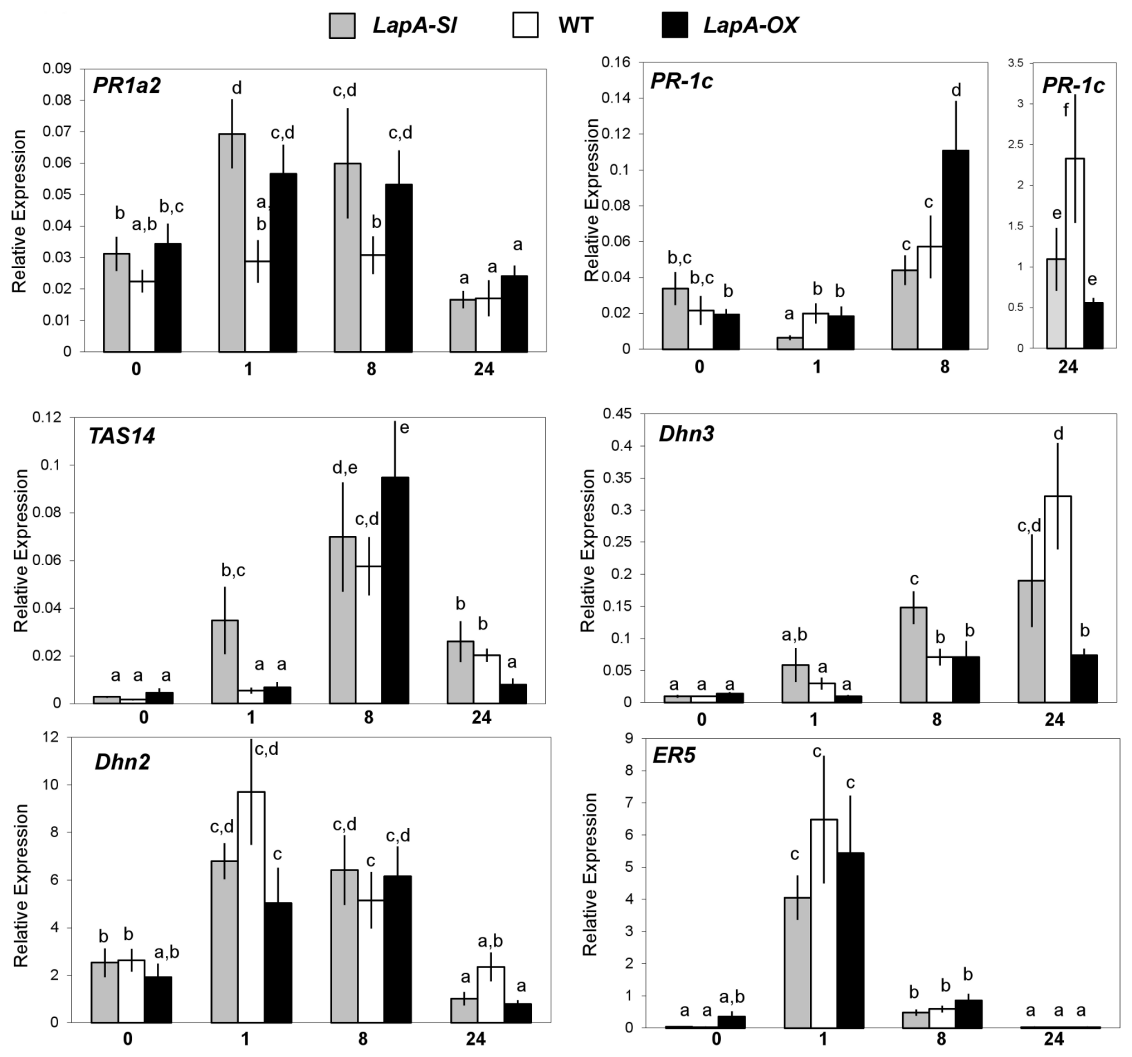
Quantitative RT-PCR analysis of selected mRNAs in leaves after wounding. Relative expression of LEA (TAS14, Dhn3, Dhn2, ER5) and *PR* (PR-1a2, PR-1c) transcripts were determined 0, 1, 8, and 24 hr after wounding in WT (white), *LapA-SI* (grey) and *LapA-OX* (black) leaves (n=3). Significant differences between transcript accumulation was determined [ANOVA, Tukey post-hoc test (p<0.05)].

 Genotype-dependent expression patterns were observed when the tomato homologs for the potato basic *PR-1* and *dehydrin* genes were studied (Figure 6). In tomato, the *PR-1* family includes five genes encoding three basic PR-1 proteins (PR-1a, PR-1b, PR-1c) with potent antifungal activity and two acidic proteins (PR-1a1, PR-1a2) (Table S12) [113,114]. Previous studies showed that *PR-1b* RNAs accumulate to high levels in response to SA, ET and MeJA, while the related *PR-1a* and *PR-1a2* are not regulated by ET nor SA [82,114,115]. While the *PR-1a and PR-1b* RNAs are pathogen induced, *PR-1a2* RNAs are not [114]. Less is known about *PR-1c* and *PR-1a1*, which are most closely related to the basic potato *PR-1* on the array (Table S12). Previous studies showed that *PR-1c* RNAs increase in response to pathogens, *PR-1a1* RNAs are not responsive to ET or SA [114]. The qPCR analyses in our study showed that the *PR-1a1* encoded a low abundance RNA and did not accumulate in response to injury in WT, *LapA-SI* or *LapA-OX* leaves (data not shown).

qPCR studies showed that *PR-1a2* RNAs did not accumulate in WT leaves after injury (Figure 6). In contrast, *PR-1a2* RNAs increased after wounding in both *LapA-SI* and *LapA-OX* plants. This pattern of RNA accumulation was surprising, since late-wound response transcripts show reciprocal responses in *LapA-SI* and *LapA-OX* lines [25]. This unanticipated pattern of gene expression could have one of two explanations. First, since both *LapA* and *LapN* are silenced in the *LapA-SI* lines and only *LapA* is over-expressed in the *LapA-OX* lines [25,116], the non-wound induced, rare class *Lap-N* could contribute to these RNA patterns. Alternatively, differences between the genotypes may suggest multiple or more complex roles for LAP-A in the signaling network that controls *PR-1a2* expression. 

LAP genotype-dependent differences in *PR-1c* gene expression were also observed. In WT plants, the *PR-1c* transcripts increased by 8 hr after wounding and were at highest levels by 24 hr (Figure 6). In the *LapA-OX* line, *PR-1c* RNAs accumulated more rapidly than in WT leaves (8 hr) and *PR-1c* RNAs either declined more rapidly or did not reach the levels observed in WT leaves by 24 hr. In contrast, in *LapA-SI* leaves, the *PR-1c* RNAs initially declined (1 hr) and did not reach the levels seen in *LapA-OX* (8 hr), or WT (24 hr) plants after injury. These data again suggest that LAP-A regulation of the *PR-1* gene family is complex.

*TAS14* is a well-characterized dehydrin from tomato that is regulated by water deficit, salinity, abscisic acid, and mannitol and is a late embryogenesis-abundant (LEA) protein [82,117,118]. The microarray suggested that *TAS14* was a 0-hr gDEG and its RNAs were injury induced (Table S11). Although *TAS14* was not verified as a 0-hr gDEG, it displayed a *LapA* genotype-dependent pattern of RNA accumulation after wounding (Figure 6). *TAS14* RNAs accumulated more rapidly in the *LapA-SI* than WT or *LapA-OX* plants and declined more slowly than in *LapA-OX* plants. These data suggested that *LapA-SI* line was primed to express *TAS14*.

 Because *TAS14* was differentially regulated in the *LapA-SI* line, additional tomato dehydrins (*Dhn2, Dhn3, Dhn4*) and LEA (*ER5, LEA-like*) genes were identified and their wound responsiveness and LAP-dependence was determined (Table S12; Figure 6; *Materials & Methods*). *TAS14* and *ER5* are currently the only characterized LEA genes in tomato [42,118]. *Dhn4* and *Lea-like* RNAs were not detected in healthy or wounded WT, *LapA-SI* or *LapA-OX* leaves by RT-PCR and qPCR, respectively (Table S11; data not shown). While the microarray predicted that the *Lea-like* RNAs would increase after wounding, the potato *LEA-like* EST array signal was likely due to cross-hybridization of the abundant, wound-induced *ER5* RNAs; *ER5* and *Lea-like* had 85% nucleotide sequence identity (Table S3). Consistent with the microarray data (Table S3), qPCR analyses showed that *Dhn2* and *ER5* RNAs peaked 1 hr after wounding (Figure 6). For *Dhn2* and *ER5*, whose transcripts accumulated rapidly after injury, there was no significant difference in RNA levels between the *LapA* genotypes. 

 Similar to *TAS14*, *Dhn3* transcripts displayed a genotype-dependent pattern of expression that altered the timing and abundance of transcripts (Figure 6). After injury, *Dhn3* RNAs accumulated more rapidly in the *LapA-SI* line relative to WT and *LapA-OX* plants. In addition, *Dhn3* RNAs were at lower levels in the *LapA-OX* line relative to WT and *LapA-SI* lines. Collectively, these data indicated that LAP-A negatively regulated the Dhns (*TAS14* and *Dhn3*) that are part of the late-wound response in tomato and not the Dhns/LEAs that are induced early (*ER5* and *Dhn2*). This temporal-specific regulation is consistent with LAP-A’s regulation of late, but not early, wound-response genes that were previously reported [25]. However, unlike other LAP-A regulated late-wound response genes, LAP-A is a negative regulator of *TAS14* and *Dhn3* rather than a positive modulator.

 Plant Dhns are a distinct subgroup of LEAs that are characterized by the presence of amphiphilic K regions and have a protective role in osmotic stress [119]. While the exact mechanism of Dhn action has remained elusive, Dhns may serve as molecular chaperones to enable refolding of partially denatured proteins. Given the fact that LAPs are both aminopeptidases and potent molecular chaperones *in vitro* [28], it is intriguing to speculate that LAP-A serves as a chaperone after wounding. In this scenario, *Dhn* gene expression increases in the *LapA-SI* line to compensate for the lack of LAP-A’s molecular chaperone activity. 

## Discussion

 In this study, tomato RNAs were hybridized to a TIGR potato 10-K cDNA array to determine the differential accumulation of RNAs after wounding. While cross-species hybridization (CSH) has been used in many studies, its results should be interpreted carefully [55]. Many studies have shown that CSH can lead to lower signal to noise ratios and this is correlated with the degree of sequence divergence between the probe species and transcript species orthologs [54,55]. This can be seen in species with even 1% sequence divergence and has been demonstrated CSH studies between potato and tomato, which have ~8% sequence divergence [54,120-122]. Lower signals lead to fewer DEGs being identified compared to other methods such as species-specific hybridization (SSH). Secondly, CSH may be more prone to signals being the result of cross-hybridization with RNAs from closely related gene family members [120]. These two factors make identification of the true target transcripts more challenging for CSH experiments, even in species as similar as potato and tomato.

 Despite these limitations, important insights can be gleaned from CSH studies. For example, this study was the first comprehensive look at transcript regulation at early times after wounding in tomato. While some of the classical early wound-response genes were not detected until 8 hr after injury, the overall transcript responses to injury strongly correlated with previous wound and JA treatment studies [18,19,21]. In addition, the array detected many other stress and metabolism pathways were regulated early and enhanced late after wounding. These findings are consistent with *Arabidopsis*, which shows rapid and early induction of a wide array of genes after wounding [10,14]. This is also the first study to comprehensively compare local and systemic transcript regulation after wounding in tomato. Consistent with other studies, systemic responses are more delayed than local responses [19,25]. However, by 8 hr, there is a large overlap between local and systemic responses, suggesting that complex metabolic responses beyond core defenses occurs even in unwounded tissue.

 Consistent with other studies, the most dramatic response to wounding was a down-regulation of photosynthesis. This was not only seen as a reduction in photosynthesis- associated nuclear gene transcripts, but also in the repression of plastid ribosomal protein RNAs. Several biogenic retrograde signals have been implicated to control nuclear gene expression during light-regulated development and after disruption of plastid translation including two tetrapyroles (Mg-Protoporphrin IX and heme) and a carotenoid oxidation product (β-cyclocitral) [123-128]. Since the suppression of photosynthesis-associated nuclear genes occurred in all three genotypes used in this study (WT, *LapA-SI* and *LapA-OX*), our data indicate that this is not a LAP-A-dependent event in wounded tomato leaves. Therefore these biogenic signals are unlikely to be “the” LAP-A-dependent signal. 

 Mechanical wounding is the source of other stresses beyond cell damage including local dehydration and susceptibility to opportunistic pathogens. Therefore, consistent with other studies, wounding in tomato induced a wide array of signaling pathways including those involved with desiccation and pathogen defense including up-regulation of genes encoding transcription factors (WRKYs and TGA2), antimicrobial PR-1s, and enzymes involved in cell wall strengthening via the up-regulation of lignins and polyamines, as well as the down-regulation of genes encoding plastid-localized ROS detoxifying enzymes. Unfortunately, the array did not provide deep coverage of genes that are sentinels of the multiple hormone pathways that are integrated for defense and wound responses. Despite this limitation, the array data suggested that there are complexities in GA signaling that are yet to be revealed.

 Previous studies have shown that LAP-A modulates the levels of late wound-response RNAs (*Pin I, PinII*, and *PPO*) in tomato [25]. The study presented here demonstrates for the first time that LAP-A controls wound responses earlier than previously recognized. Importantly, LAP-A serves as both a positive and negative regulator of gene expression after injury. Microarray analysis identified cohorts of stress-response genes that displayed altered expression programs after wounding in *LapA-SI* plants. In the future, high-throughput sequencing methods will be able to more accurately quantify the temporal changes in gene expression in WT, *LapA-SI* and *LapA-OX* plants and more precisely identify the full spectrum of LAP-A targets.

 Finally, two new groups of LAP-A-modulated genes were identified through studies characterizing the tomato *PR-1* and *Dhn/LEA* gene families. While *PR-1c* and *PR-1a2* transcript levels were regulated by LAP-A, the mechanism of regulation is likely to be complex. This is based on the fact that unlike the late wound-response genes [25], the changes in *PR-1c* and *PR-1a2* RNAs in the *LapA-SI* and *LapA-OX* line were not reciprocally regulated. Since regulation of *PR-1c* and *PR-1a2* has not been intensively investigated, further dissection of the signaling pathway(s) required for *PR-1c* and *PR-1a2* transcript accumulation will be a fruitful area of investigation. 

 These studies also revealed that LAP-A is a negative regulator of two *Dhn* genes (*TAS14* and *Dhn3*) that were expressed in the late phase of wound signaling and, in this case, reciprocity in the *LapA-SI* and *LapA-OX* plant phenotypes was observed. These data indicate that LAP-A has a broader role in regulation of gene expression after injury – serving as a positive regulator of the late branch of wound signaling (ie., *PinI, PinII*, and *PPO*) and a negative regulator of *Dhns* (*TAS14* and *Dhn3*) expressed during this same time frame. Given the recent finding that LAPs are both aminopeptidases and molecular chaperones [28], future research will focus on identifying whether LAP-A peptidase and/or chaperone activities mediate these critical roles in plastid to nucleus communication that regulates defense signaling. Future studies will also determine the nature of the LAP-derived retrograde signal. It is of interest to determine if the LAP-derived signal is similar to the operational retrograde signals used in *Arabidopsis* biotic and abiotic stress responses or if a novel signaling molecule is involved. 

## Materials and Methods

### Plant materials and growth conditions

*Solanum lycopersicum* L. UC82 (wild-type), *P35S:LapA-SI*, and *P35S:LapA-OX* were previously described [25]. Plants were grown in a growth chamber with an 18-hr (28°C)/6-hr (24°C) light (300 μE)/dark cycle as described [82].

### Wound treatments

 Three- to four-week-old plants were used in the wounding time-course studies. Plants were wounded by crushing the distal end of each leaflet with a pair of needle-nosed pliers. Two lower leaves were wounded (local response) and all leaflets (typically 6-8 leaflets) from wounded and the two apical leaves (systemic response) were collected at designated times. The leaves of five plants at each time point were pooled together for RNA extractions. This experiment was repeated three times for microarray analysis. Experiments were repeated additional three times for RT-PCR or qPCR analyses. 

### RNA isolation for microarray and real-time PCR analysis

 RNAs were extracted using a hot phenol method as previously described [29]. RNA for microarray analysis was further purified using the SV Total RNA Isolation System (Promega, Madison, WI USA). RNA was quantified and 260/280-nm absorbance ratios were measured using a Nano-Drop ND-1000 spectrophotometer. RNA quality was also ensured by checking for the presence of intact rRNA bands by 1.5% formaldehyde gel. 

### Microarray hybridizations, scanning and data acquisition

 RNAs were hybridized to TIGR potato 10,000-clone version 3 cDNA microarrays (http://www.tigr.org/tdb/potato/microarray_comp.shtml). At the time these experiments were performed, the TIGR arrays were an economical option for exploring the tomato transcriptome since the arrays and services were subsidized by the NSF. All steps of microarray processing to obtain raw data (cDNA production, cDNA labeling, and microarray hybridization were carried out by the TIGR Expression Profiling Service according to published methods (http://www.tigr.org/tdb/potato/microarray_SOPs.shtml ). A reference design hybridization strategy was used with WT (0-hr) being the reference RNA [41]. The reference RNA was a pool from five WT 0-hr RNAs. RNAs from wounded or unwounded leaves labeled with CY3 and the reference RNA labeled with CY5 were co-hybridized to the potato cDNA array. Three dye bias experiments were performed in which WT 0-hr RNAs were labeled with CY3 and co-hybridized with the CY5-labeled reference RNA (WT 0-hr RNAs from a different pool). After normalization, no dye bias was detected on these experiments (*p*-value<0.05). Raw data and metadata spreadsheets have been deposited in NCBI's Gene Expression Omnibus (GEO) [129]. These data are accessible through the GEO series accession number GSE49419 (http://www.ncbi.nlm.nih.gov/geo/query/acc.cgi?acc= GSE49419).

### Image and data analysis

 Spot data was extracted using GENEPIX (ver. 5.0 Pro: Axon Instruments, Union City, CA, USA) at TIGR. Data output obtained from GENEPIX are publicly available and can be downloaded through a database maintained at the TIGR Web site (http://www.tigr.org/tigr-scripts/sgedb/studies_SGED.pl). A series of quality assessment routines were applied to the microarrays. This included ratio-intensity plots (also known as MA plots) within and between arrays and print tips, as well as distribution plots of intensities. Normalization and differential gene expression analysis steps were performed in R (http://www.r-project.org) using the LIMMA package [118]. Here the data sets were normalized within arrays using the print-tip loess method. Background correction was omitted because it added too much variation to the data. In addition, arrays were normalized between arrays using quantile normalization as described in [130]. Within-array duplicate spot correlations were calculated and duplicate spots were weighted using the duplicateCorrection function of the LIMMA package [131]. 

 Statistically significant differentially expressed genes (DEGs) among sample treatments, each with three biological replicates, were identified using the empirical Bayes method implemented in the LIMMA package [132]. To control the false discovery rates (FDR) of candidate DEGs, their raw p-values were adjusted for multiple testing using the Benjamini & Hochberg method [133]. As confidence threshold for DEGs we used a log_2_ fold change (|FC|) ≥ 0.8 and an adjusted *p* < 0.05. 

### Quantitative PCR analysis

 Selected potato cDNA sequences indicated as differentially expressed in microarray analysis were aligned with tomato sequences using the Basic Local Alignment Search Tool (BLASTN) and the Sol Genomics Network (SGN) combined tomato Unigene database (01-24-10; http://solgenomics.net/) [134]. cDNA clones of tomato genes with high nucleotide sequence identity to the potato ESTs (expectation (E) value < 1e-30) were obtained from Boyce Thompson Institute (BTI) and were confirmed by DNA sequencing at the University of California Riverside’s Institute of Integrative Genome Biology Genomics Core. 

 Total RNA was DNase treated using RQ1 RNase-Free DNase (Promega, Madison, WI). RNase H^+^ iScript reverse transcriptase (Bio-Rad Laboratories, Hercules, CA) was used to perform reverse transcription (RT) according to the manufacturer’s instructions. cDNAs were diluted 10 fold in water for qPCR analysis. All mRNA levels determined by qPCR analysis were normalized with the tomato translation EF1a, ubiquitin (Ubi3), and housekeeping gene 4 (HKG4; a hypothetical protein) as previously described [25]. Gene-specific primers were designed to amplify unique regions of the tomato genes of interest compared to highly related gene family members based on the sequences obtained from tomato cDNA clones. Primers were designed using Primer3 [135] and annealing temperatures and efficiencies were determined experimentally. Primer sequences, annealing temperatures, and Unigene numbers used are listed in the Table S13. 

 For preliminary screening of 0-hr gDEGs, semi-quantitative RT-PCR was performed as described in [136]. Primers were optimized using plasmid DNAs as templates and confirmed by amplifying tomato genomic DNA. RT-PCRs with cDNAs were amplified for 23-29 cycles and normalized to eIF4 (SGN-U581466) (26 cycles). For more quantitative measurement, qPCR reactions were performed in triplicate using iQ SYBRGreen Super-mix (Bio-Rad) and data was analyzed using the real-time PCR miner program [137] according to [25]. Averaged Ct values and averaged efficiencies of replicate samples were used to calculate mRNA levels of reference genes. Individual Ct values of replicate samples and the individual efficiencies of replicate reactions were used to calculate mRNA levels of wound-inducible genes. For each wound-inducible gene, RNA levels at each time point were normalized against the geometric mean of the RNA levels of the three reference genes at each time point. 

### MapMan analysis

 The ratios obtained from the microarray analysis were imported into MapMan Software [44]. Annotation and functional characterization was assigned using Stu_TIGR.m02 August07 [43]. Annotation for selected genes was confirmed by BLAST searches of the potato cDNA sequences against the TIGR tomato EST database (http://www.tigr.org/) using BLAST. MapMan was used to perform a Wilcoxon rank sum test to determine metabolic pathways that were the most differentially regulated as described in [45]. 

## Supporting Information

Figure S1 **Local and systemic changes of putative 0-hr g*DEG* RNAs after wounding.** FC based on microarray analysis of *LapA-SI* 0-hr g*DEG* RNAs 0, 1 and 8 hr after wounding in *LapA-SI* and WT local and systemic tomato leaves.
(TIF)

Table S1 **MapMan BIN assignment of DEGs after wounding in WT and *LapA-SI* leaves.**
(XLSX)

Table S2 **Gene expression in WT and *LapA-SI* lines after wounding of all genes on potato array.**
(XLSX)

Table S3 **Differentially expressed stress-responsive genes (BIN20) after wounding of WT and *LapA-SI* leaves.**
(XLSX)

Table S4 **Differentially regulated genes after wounding in WT and *LapA-SI* lines represented by multiple clones on TIGR 10-K cDNA microarray.**
(XLSX)

Table S5 **Differentially expressed photosynthesis and tetrapyrole synthesis genes in response to wounding in WT and *LapA-SI* leaves.**
(XLSX)

Table S6 **RNA and protein metabolism genes differentially regulated after wounding in WT and *LapA-SI* tomato leaves.**
(XLSX)

Table S7 **General metabolism genes differentially expressed in WT and *LapA-SI* leaves after wounding.**
(XLSX)

Table S8 **Differentially expressed hormone metabolism and response genes after wounding in WT and *LapA-SI* leaves.**
(XLSX)

Table S9 **Top 100 differentially expressed genes at 0 hr after injury of *LapA-SI* leaves relative to WT plants.**
(XLSX)

Table S10 **Top 100 differentially expressed genes at 8 hr after injury of *LapA-SI* leaves relative to WT plants.**
(XLSX)

Table S11 **Differentially expressed genes in *LapA-SI* leaves before wounding.**
(XLSX)

Table S12 **Putative 0-hr gDEG homologs and gene family members.**
(XLSX)

Table S13 **Primers and their conditions used for RT-PCR or qPCR analysis.**
(XLSX)
