# Supplementary figures and images for: Microarray Analysis of Tomato’s Early and Late Wound Response Reveals New Regulatory Targets for Leucine Aminopeptidase A
Source: PLoS One. 2013 Oct 24;8(10):e77889. doi: 10.1371/journal.pone.0077889 (PMC3812031; doi:10.1371/journal.pone.0077889)

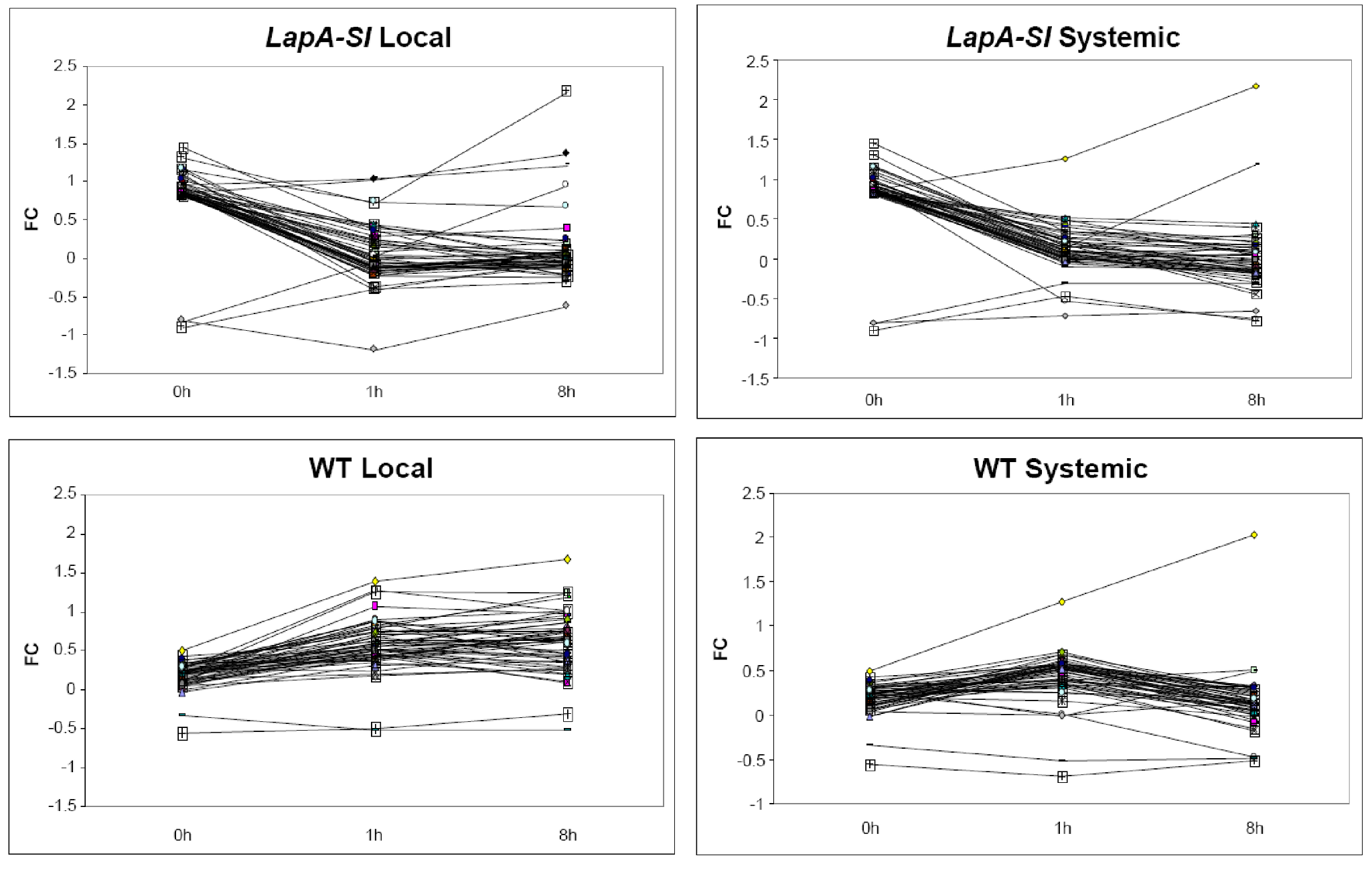

Supplement: Figure S1 — Local and systemic changes of putative 0-hr gDEG RNAs after wounding. FC based on microarray analysis of LapA-SI 0-hr gDEG RNAs 0, 1 and 8 hr after wounding in LapA-SI and WT local and systemic tomato leaves. (TIF) [file pone.0077889.s001.tif]
